# Routines, Time Dedication and Habit Changes in Spanish Homes during the COVID-19 Lockdown. A Large Cross-Sectional Survey

**DOI:** 10.3390/ijerph182212176

**Published:** 2021-11-19

**Authors:** Miguel Ángel Navas-Martín, José Antonio López-Bueno, Ignacio Oteiza, Teresa Cuerdo-Vilches

**Affiliations:** 1 Escuela Nacional de Sanidad, Instituto de Salud Carlos III (ISCIII), 28029 Madrid, Spain; manavas@isciii.es (M.Á.N.-M.); jalopez@isciii.es (J.A.L.-B.); 2 Instituto de Ciencias de la Construcción Eduardo Torroja, Consejo Superior de Investigaciones Científicas (IETcc-CSIC), 28033 Madrid, Spain; ioteiza@ietcc.csic.es

**Keywords:** housing, confinement, coronavirus, household, gender, routine, Spain, questionnaire, built environment

## Abstract

Many countries chose to establish social distancing as lockdowns after the COVID-19 outbreak. Households had to adapt their day-to-day lifestyles to new circumstances, affecting routines and time dedication to tasks. This national study was carried out to find out how the confinement by COVID-19 affected Spanish households on the perceived habit changes during this period, in relation to their socio-demographic characteristics and household composition. An online questionnaire was launched during the COVID-19 lockdown, from 30 April to 22 June 2020. Descriptive statistics were analyzed, stratified by gender, on time dedication, routine, home leaving, and habit change variables. Chi-square tests were used to explore the relations of significance with socio-demographic characteristics and home composition. All contrast analyses were performed for a 95% confidence level (significance considered for *p* < 0.05). In total, 1673 respondents participated from different age groups, educational level, employment status and household composition. Sixty percent of respondents maintained their routines. A third tried to establish a new one, being related to women, young people, not a university student, and living with others, including minors. Regarding dedication to tasks, adults aged 35–54 years, with more cohabitants, especially women, devoted themselves intensively to the home or to care, while those under 35 were dedicated more to rest, leisure, television or reading. People with university studies were more related to teleworking. The frequency of going outside was related to gender, age, educational level and living with elders, specifically for grocery shopping and taking out garbage. Changes in habits, routines and time dedication in confinement were strongly linked to the sociodemographic and coexistence conditions in Spanish homes. The greatest impacts were suffered by women, people with children, and adults between 35–54 years of age, especially on care and domestic chores.

## 1. Introduction

The SARS-CoV-2 coronavirus pandemic has swept the world since it emerged in late 2019 in China. In Europe, the first cases were confirmed on 30 January 2020, while more than 7800 cases were confirmed worldwide [1]. On 11 March, the World Health Organization decided to raise the incidence of COVID-19 to the degree of pandemic [2].

In Spain, the first case was detected on 31 January [3]. Given the imminent incidence in Europe, and three days after the declaration of a pandemic, the Spanish government declared a State of Alarm [4], by which all nonessential movements were restricted, and activity at the national level, using confinement as a measure of prevention and containment of the transmission of the disease [5]. This included the closure of schools and other educational centers [6], as well as teleworking as far as possible [7]. With the indefinite suspension of these activities, homes became fully occupied for a full and permanent period for one month and a half. After this period, the Spanish government established a transition plan toward the new normal. They established four phases (phase 0: de-escalation preparation, phase I: initial, phase II: intermediate, phase III: advanced) of de-escalation conditioned by the epidemic situation in a progressive way [8], with phase I being the most restrictive and the last phase without restrictions.

Progressively, phases became more flexible and allowed timetables and activities abroad, but not in a homogeneous way. The uneven de-escalation plan began on the islands and continued by province, for partial mobility and attendance to certain priority needs. Thus, overlapping degrees of de-escalation throughout the territory happened, according to the public health situation of each area. On 11 May 2020, only 54% of provinces (27) were in phase I [9]. After one month, on 18 June, 82% of them belonged to an advanced phase (41), and only 8% (four provinces) were in the new normal [10]. Finally, the national State of Alarm ended on 21 June 2020 [11].

According to the National Institute of Statistics, between January and May 2020, there were 32,652 deaths due to COVID-19 and 13,032 suspected of dying due to symptoms compatible with the disease. The month with the most deaths from COVID-19 and suspected of COVID-19 was April, with a total of 26,305 deaths [12].

Regarding the notified cases with a positive diagnostic test for active infection, during the data collection period (30 April to 22 June), 73.9% of positive cases (21,194) were between 30 April and 31 May, while the rest of the positive cases was at 26.1% (7480) between 1 June and 22 June [13].

**Figure 1:**
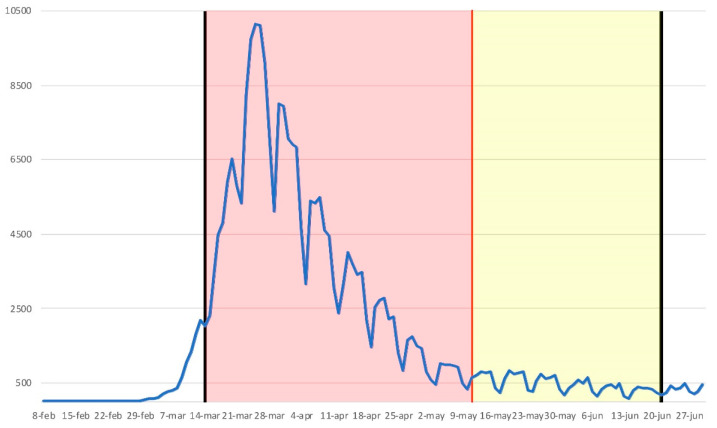
Number of daily COVID-19 diagnosed cases within the first wave of the pandemic in Spain. Grey lines delimit the State of Alarm. Red shadow shows the total-confinement period, and yellow shadow shows the de-escalation period once cases decreased enough.

The daily diagnosed cases of COVID-19 in the first wave of the pandemic (which basically coincided with the national State of Alarm in Spain) are shown in Figure 1. In it, the differentiated sub-periods are superimposed as total confinement (the entire population locked up 24/7, except for specific cases of force majeure or essential activity, in Figure 1, defined by a red shading) compared to the de-escalation period, which, as mentioned, had various sub-stages, some of them occurring simultaneously by territorial areas, according to the level of transmission of COVID-19 (in Figure 1, period with yellow shading).

As shown in Figure 1, the national State of Alarm helped to drastically stop the transmission of the disease. This social norm, of an imperative nature, obtained the desired result, especially during total confinement. Once certain epidemiological requirements were met at the territorial level by provinces, these advanced de-escalation stages, which began on May 11, were conditioned to such requirements. However, as it occurred unevenly in the national territory, it generated different degrees of social mobility and performance of activities, depending on the spread of the disease by geographical area.

## 2. Literature Analysis

### 2.1. Habits and Routine Behavior

To understand what a habit is, and how it is produced, there are many references from various disciplines, such as psychology or sociology, included within the behavioral sciences. By way of summary, from the sociological field, the best known or accepted ones can be reviewed—among them are those related to American pragmatism during the first third of the 20th century, or Bourdeau’s position at the end of the same century.

At the dawn of the 20th century, Dewey defined habit as the engine of human action, which is influenced by group customs. Human actions must be learned; thus, habits bring together a series of ordered actions that provide comfort, ability and interest, once a certain habit is generated, to those who carry them out. Exercising the habit does not mean excluding thought, although it does channel it, exercising it in the spaces between habits [14].

Bourdeau developed the theory of “habitus”, starting from Aristotelian concepts, Weber and (post)-husserlian phenomenology [15]. The habitus starts from a sociological, historical, and structuralist point of view, beyond naturalistic or mechanistic approaches. According to him, individuals (their bodies) have a natural predisposition to acquire non-natural, arboreal abilities, although these must obey an external stimulus under certain circumstances. Through habitus, a system of “durable and removable” predispositions is formed. Bourdieu refers to “agents”, endowing them with the skills of invention and improvisation. Habitus schemes allow constant adaptation to partially modified contexts, being able to meaningfully deconstruct a certain event by anticipating certain tendencies and behaviors that come in turn from all isomorphic habitus, immediately related [16].

Habitus are therefore “acquired schemes operating in the practical state as categories of perception and appreciation or as principles of classification, as well as organizing principles of action”. Unlike habit, repetitive, mechanical, automatic, and more reproductive than productive, the habitus is considered as something potently generative [17].

### 2.2. Habit Changes and Adaptive Routines in Times of Emergencies and Disruptive Events

Human agency has been defined, but there are other questions that analyze when, where and under what conditions habits prevail or, on the contrary, when internal reflection is prioritized [18]. For Camargo, habits and reflection depend (also) on people’s beliefs and can punctually differ if satisfaction is perceived or not. If people perceive truthfulness in their beliefs, they will act out of habit [19]. Social satisfaction is not always born from empirical or verifiable verification, but from the joint belief of different groups. When this does not occur, there is no consensus, and critical episodes can arise.

This reflection would involve questioning one’s own belief, either discarding it, or trying to argue it to support it. Therefore, habit arises by satisfying belief, whether it is verifiable or not. These habits, once they lose their intentional character, are inscribed in the background [20], in what others call routines.

In a simplified way, the power of human action or active response, also called “agency”, depends on the state of the individual’s beliefs, on the changes in the social structure (political, economic), and on those that are symbolic–cultural. In modern societies, where pluralism occurs, its members are more reflective [19].

Governments have the ability to effectively and quickly intervene and reform society and ways of life; society has supported this intervention in the service of social threats, such as health emergencies. However, not all threats to public health are supported in the same way, nor are they addressed in the same way by society. If the threat is immediate and direct, the social response is driven by necessity, and therefore it will be more radical and urgent (they are closer to people). Furthermore, if risks can somehow be mitigated in a relatively social “easy” and understandable way, it will be easier to assume. In addition, the temporary provision to execute these actions, and abide by the rules, is decisive to obtain a social response or another. As opposed threats and their social understanding in this sense, COVID-19 and climate change could be cited, for instance [21].

### 2.3. Habits, Routines and Time Dedication in Times of COVID-19

During the COVID-19 pandemic, many studies have been interested in knowing the social habits and the changes that they have experienced when physical distance was imposed.

Some of these studies delved into the effects on the population of childcare [22], remote working [23], work–family reconciliation [24], food insecurity and dietary quality [25], sleep quality [26], leisure and sport [27]. In turn, these studies have been able to focus on the general population [28], as well as on specific segments, such as the elderly [29,30], chronic patients [31], adolescents [6], women [32], or in directly affected unions, such as health workers [33], teachers [34], etc.

Ultimately, these studies addressed the consequences on people’s physical, psychological, and emotional wellbeing. This situation altered the mental health of the population, causing situations of anxiety, stress and depression [35].

In many of these processes of wellbeing or lack thereof, the perception of the dwelling or its spaces also played a role, and therefore the adaptability it offered in the face of changes in activities or habits was required by circumstances [36,37]. However, studies where people reported general occupational engagement during lockdown, compared to the pre-pandemic situation, and their perception of altered habits, going out of the house, and changes in their daily life in which the home is the center of activity, were not common [38]. Other studies focused on socio-demographic characteristics such as gender [39], or household composition [40], without a generalized picture of Spanish households.

The aim of this study has a double mission: (1) to describe the changes in habits, routines, going out of the house, and time dedication that occurred in Spanish households during the social confinement produced by the COVID-19 pandemic, together with the associated factors (sociodemographic and of cohabitants), and (2) to identify which of these factors have affected those changes in the daily lives of the residents. Through this study, the authors hope to help unveil the reality of the residents in order to contribute to decision making and the creation of strategies and contingency plans, related to the social management of time. In addition, they hope to help to create support networks and the design or cession of common spaces where people can alleviate certain burdens that have generated great tension, while guaranteeing safety conditions against COVID-19 transmission.

## 3. Materials and Methods

This cross-sectional study was carried out for the Spanish population between 30 April and 22 June, the period covered by the State of Alarm decreed by the national government on 14 March 2020 [4]. Its purpose was to find out the reality of Spanish households during the period of confinement. This study was funded by the Spanish National Research Council (CSIC), obtaining the approval of its Ethics Committee, with report number 057/2020.

Household representatives participated anonymously and independently in an online forum. The topics addressed in the questionnaire covered aspects of changes in habits, temporary dedication to certain tasks, routines, and going out of the home for various reasons.

Confinement, the object of study as a phenomenon, made contact with potential participants difficult. For this reason, an online, anonymous, self-completed questionnaire was established. The target population was selected with a non-probabilistic sample by convenience. Using the web scraping technique, the e-mail addresses of numerous groups, such as neighborhood and cultural associations and town councils, were obtained, to which the information sheet for the study and the web link to the questionnaire itself were sent. The purpose of this was that these groups would facilitate the contacting of a greater number of people, and a wider distribution throughout the national territory. Social networks, institutional websites and instant messaging applications were also used to expand the number of participants. Informed consent was implicitly understood by accepting access to the questionnaire after reading the information provided at the beginning, which also referred to the objectives of the study and its researchers and the organizing entity. All the information given, as well as consent, was approved by the aforementioned ethics committee.

The digital platform for collecting the results of the online participation was SurveyMonkey^®^. A database was then generated to organize and work with the information obtained.

The original self-administered questionnaire contained 58 questions, combining both numerical and categorical responses as well as Likert-type. This questionnaire is based on other previously validated questionnaires, collected by specifically related regulations [41] or applied in studies and accepted by the scientific community as a common way of collecting data on cohabitants in studies on dwellings [42,43,44,45]. This questionnaire was previously carried out among ten people to ensure its readability, comprehension, compliance, such that improvements could be made in order to launch it to the target audience. To ensure that there were no duplicate questionnaires and that there were no inconsistencies, measures were taken such as stating in the initial informed consent that they were the only representative of the household completing the questionnaire, as well as detecting and eliminating recurrent duplications in responses and other inconsistent data. This study focuses on those questions related to changes in household habits, the time dedication to tasks in the dwelling, going outside, and the establishment of routines during confinement.

Six categories were distinguished for this analysis, with their corresponding variables used (Table 1).

The study is based on the responses obtained from the participants at the national level, where a descriptive analysis was carried out, stratifying the responses obtained by gender in the case of the socio-demographic variables, in addition to a bivariate analysis applying the Chi-square test.

In relation to the variables of establishment of routines, dedication to tasks, frequency of leaving home, and change of habits, a bivariate analysis was applied to check for possible statistically significant relationships with socio-demographic variables and cohabitants, also using the Chi-square test. All contrast analyses were performed at a 95% confidence level (significance if *p* < 0.05).

## 4. Results

For this analysis, a total of 1673 valid responses were counted, of which 62.5% were women, 80.5% had a university education, 47.9% worked for the administration and 93.2% were of Spanish origin.

### 4.1. Characteristics of the Representative Members of the Participating Households

Table 2 shows the socio-demographic characteristics of the study participants, representatives of each household. At the beginning of the questionnaire, it was requested that only one member of the household complete the questionnaire, in order to ensure a one response–one household correspondence. These responses were stratified by gender.

Regarding cohabitation at home during confinement, 22.7% lived alone, 27.8% with another person and 49.5% with at least two other persons in the same household. In addition, 36% lived with children under 18 and 14.6% with people over 65. The cohabitation variables were not significantly related to gender but were significantly related to age. Being over 55 was related to living alone (29.3%) and being between 25 and 54 to living in households with more than two cohabitants (58%). People over 55 had a significantly higher proportion of cohabitation with those over 65 (35.2%) than younger participants. In contrast, the population with the significantly higher percentages of cohabiting with children was in the 35–54 age group.

Level of education and country of origin were statistically significantly, relating to the cohabitation variables. People with university studies lived proportionally more alone (23.9%) than those without them (17.3%), and conversely, non-university graduates tended to live more in households with more than two cohabitants (54.8%) versus university graduates (48.1%). People of non-Spanish origin lived more in households with two members (37.1%) than those of Spanish origin (27.3%), and the latter lived more in households with two or more people (50.1%) than the former (38.1%). Civil servants (11.2%) and those who were self-employed or entrepreneurs (14.8%) were significantly more likely to live with people over 65 than those who were employed (5.3%).

### 4.2. Establishing Routines

Half of the surveyed population (50.1%) stated that, during lockdown, they maintained their previous usual routines, albeit in a more flexible manner. It is remarkable that “establishing new routines” is related to changing habits and/or the schedule in which they are organized and executed by individuals, as a sequence of habitual actions. In this sense, 31% tried to establish a new routine, 9.1% made no changes to their previous routine, and 9% maintained no routine at all. This variable was related to several socio-demographic and cohabitation characteristics of the respondents in a statistically significant way (*p* < 0.05). For example, women were more likely to establish new routines than men (32.6% vs. 28.5%) and less likely to cope with confinement without routines (7.2% vs. 12.1% in men). Young people (18–34 years) either tried the most to establish new routines (36.9%) or established them the least (11.6%). There were significantly more people who did not establish routines among non-university graduates (12.2%) than among university graduates (8.3%), and they also tried more to create new routines (38.9% vs. 29.2% in the university-educated population). People living alone were more likely to maintain their usual routines (12.4%) than those living with others (8.1%) and were less likely to make those routines more flexible (45.8% vs. 52.6% of those living with others). Living with people over 65 was not related to the establishment of routines, while living with children was: people living with children were more likely to establish new routines (33.8%) than those who did not (29.2%) and less likely to maintain the same routines (6.9% vs. 10.4%).

### 4.3. Time Dedication to Different Tasks

Table 3 shows the amount of time dedicated to tasks in confinement, according to socio-demographic and cohabitation characteristics.

Time dedication to certain tasks was scored on a Likert scale from 1 to 5, where 1 was no time and 5 was a lot of time. Figure 1 shows the frequency distribution of dedication to tasks during confinement, classified as low dedication (scores 1 or 2), medium (scores 3) and high (scores 4 or 5). Table 2 shows the percentages of high dedication to each of the tasks studied, according to socio-demographic and cohabitation variables. People under 35 years of age related to having a higher dedication to rest, leisure and watching TV or reading, while people between 35 and 54 years of age had a higher dedication to home or care. People over 55 were least likely to be engaged in teleworking or tele-study. Being a woman was associated with a higher commitment to care and home. People without a university education were more likely to spend more time resting, doing housework and watching TV or reading than those with a university education, but less time teleworking or tele-studying. People living with more than one other person were the most likely to be engaged in housework and care work, and the least likely to be engaged in resting and watching TV or reading. Living with older people was associated with more time spent watching TV or reading and less time spent on teleworking or housework. In contrast, living with children was associated with less time spent resting, watching TV or reading, and more time spent on household chores or care. Origin and employment status did not show a statistically significant relationship with engagement in these tasks.

### 4.4. Frequency of Going Outside during Confinement

During lockdown, 45.7% of the respondents hardly went outside, 38.1% went outside only occasionally and 16.2% went outside quite often. The frequency of going out to the street was statistically significantly (*p* < 0.05) and related to gender, age, educational level and cohabitation with older people. Women were the most likely to stay at home (49.3% vs. 40% of men), and men were most likely to go out frequently (19.9% vs. 13.8% of women). People over 55 stayed at home the least (37.4%, 47.2% in 35–54 year olds and 51.9% in under 34 year olds) and went out more occasionally (45%, 37.5% in 35–54 year olds and 31.6% in under 34 year olds), although there was no difference in frequent going out. People with up to secondary education went out more (never 19%, occasionally 57.1%, frequently 23.8%) than those with high school education (never 45.1%, occasionally 37.1%, frequently 17.9%) or university studies (never 46.8%, occasionally 37.8%, frequently 15.5%. Those living with people over 65 went out less frequently than the ones who did not (never 41.9%, occasionally 45.2%, frequently 12.9% vs. 46.3%, 36.9%, 16.8% respectively in those who did not live with people over 65), close to statistical significance (*p* = 0.056).

Among those who went out occasionally or regularly, the most common reasons for going out were to go shopping (85.4%) and to take out the rubbish (58.1%). Furthermore, 28.2% went out for work, 17.9% for walking the dog and 10.7% for health care visits. No statistically significant relationship was found between gender or age and going out to do the shopping, or other sociodemographic or cohabitant variables. Taking out the rubbish was only statistically significantly related to age or employment status. People over 55 years of age were less likely to take out the rubbish (50.7%) versus people between 35 and 54 (59.7%) or those under 35 (62.9%). Entrepreneurs were less likely to leave the house to take out the rubbish (30.8%) than the self-employed (49.5%), and the self-employed were less likely to go out to throw away the trash than employees (56.3%) or civil servants (61.2%). Going out to work was related to age and living with people over 65: the age group that went out to work the most was 35–54 years old (32.3% vs. 23.1% for those under 35 or 23% for those over 55); and people who did not live with older people went out to work more than those who did (29.8% vs. 19.8%).

### 4.5. Changes in Habits during Confinement

General confinement led to a number of changes in the habits of the respondents. Some of the most frequent changes are those related to everyday aspects such as work (67.6%), social relations (65.9%), leisure (56.2%) or dressing (38.1%). These changes in areas were closely related to the socio-demographic and cohabitation characteristics of the participants, as shown in Table 3. It seems that these changes affected less old people, and more women, people with university studies, people of Spanish origin, civil servants, people who live with children, and people who do not live with people over 65 years of age.

In terms of care, their related tasks and habits also underwent significant changes, such as cooking or tidying up (43.7%), cleaning (42.4%) or caring for children (32%). These changes are closely related to the characteristics of the person and their cohabitants, as shown in Table 4. Changes in care habits during confinement affected more women, people aged between 35 and 54, university graduates, people of Spanish origin, civil servants or employees, people who lived with more than one person, people who lived with children and people who did not live with people over 65.

To a lesser extent, health-related habits such as sports (53.1%), sleeping (35.1%), eating (22.5%) and slightly less, drinking alcohol (10.5%) or smoking (6.2%) were altered during the blockade. These changes were related to socio-demographic and cohabitation variables (Table 4). Having the least change in health-related habits during the lockdown was related to being over 55 or living with people over 65, while the greatest changes were related to being female. In terms of educational level, this affected the various habits differently: the population with a university education altered sports more, while it altered smoking less. There were no statistically significant differences for the variables’ origin, employment status, number of cohabitants or cohabitation with children, except for the change in smoking habits (*p* = 0.045) and living alone (9.1%), which is higher than among people living with others (6.1%).

## 5. Discussion

In Spain, total confinement lasted one month and a half, extending this period to three months with more relaxed, although controlled and gradual, measures. During all this time, the home became the place where most citizens remained (totally at the beginning). This meant that the characteristics of the dwelling conditioned the way of life in them and therefore their occupational and behavioral habits [38].

According to the results, the majority of respondents shared a household with at least two other people. In contrast, people living alone were related to being over 55 years old. This is in line with the average size per Spanish household, which is 2.5 persons, according to official data from the National Institute of Statistics. This source also indicates that people over the age of 65 comprise the most represented group living alone [46].

Considering that, in the event of infection, the main recommendation is isolation from other members of the household [45], the number of people living together is a determining factor [46]. This is also related to the size and spatial distribution of the dwelling, as the likelihood of transmission in densely populated or overcrowded households is logically much higher, as it is impossible to maintain this distance [47]. Conversely, the older they are, the more likely they are to live alone, such that support structures outside the home itself are essential to be able to know the state of these people, and to monitor and support possible needs in situations as disruptive as these [48].

While 60% of the sample stated that their pre-confinement routines were not altered, or not significantly, the rest of the sample did experience changes. The segment of the population that was most affected by this alteration was women, who had to establish new routines in confinement to a greater extent. This was associated with living with (and caring for) children or other dependents. This is related to the interaction between mothers and children, and the level of household chaos; mothers who reported the importance of routines in the lives of children in confinement for a US study rated their children’s sleep, children’s behavior, and reported less screen time [49].

The confinement situation has meant that women have been more involved in the home and in caregiving. This is in line with other countries, as in the case of Turkey, which reported in a qualitative study that women interviewed revealed greater responsibility in the home, thus consolidating traditional domestic roles [50]. This is also in line with the results reported by Eurofound, which established a generalized worsening of the gender gap throughout Europe, both because of the greater job insecurity suffered by women, and because of the role of caretakers that they have played during this confinement, as well as the need to reconcile work and family life [51]. As for women teleworkers, who have increased during confinement in order to be able to attend to all these roles [7], unequal results are reported in terms of productivity during this period. However, according to the Ellen et al. study, having meaningful goals or activities, whether imposed or self-determined, may have helped to develop greater resilience and engagement in the face of such obligations, which was positive for balancing mental health [52]. Nevertheless, this balance could easily be blurred by the uncertainty of the circumstances, which may have caused psychological disturbances and loss of well-being [53]. For the reasons mentioned above, women were the ones who stayed at home the longest, while men were the ones who went out more, either for work or to cover essential household needs.

In terms of time spent, this was unequal according to age. Those under 35 spent more time on leisure, compared to the 35–54 age group, who spent more time on housework and care. The situation of confinement has been pioneering for many, and the need to combine housework with work has been a major challenge for many families. However, in the younger age group, there are many university students, with no family responsibilities, and living with other members of the household; thus, this need would be reduced in comparison with other age groups.

Young people would also generally have no need to go outside. People over 55 years of age went out more than younger people. This may be explained by the rate of people in this age group living alone or with people over 65 years of age, thus being responsible for essential household provisioning and logistical tasks, as well as other essential tasks that involved going outdoors.

With regard to people who were highly engaged in teleworking, these were more associated with people who were qualified, university educated, and under 55 years of age. This is confirmed by previous analyses in the same research project, which associated teleworking with having a certain socio-economic status (SES) [7,54]. According to official data, teleworkers, although with a more diversified profile during the pandemic, are mostly skilled, freelancer or self-employed, with a medium-high income and a high level of education [55]. The perceived impact of the alteration of habits and the simultaneity of tasks to reconcile work and family life would condition the perception of telework, as indicated by previous analyses referred to above [7,54], which is supported by other similar studies [56].

For people over 55, and especially for those in households where there were people over 65, the most important tasks were related to leisure and distraction, such as reading or watching TV, since there were probably not proportionally greater work obligations or childcare.

As for other health-related habits, these were more altered in the case of younger people or those with university studies. Physical activity was affected for more than half of the respondents, sleep for more than one-third of the sample, and eating habits for almost one-quarter. This is confirmed by studies such as that of Kontsevaya et al., which justified the disturbance of rest in turn by changes in mealtimes, teleworking or increased use of screens [56]. Yet, this disturbance does not necessarily lead to less rest, as a national study found that people who slept 6 h or less per day decreased during confinement [57].

Taking into account that the literature supports the ability to adapt their lives to a disruptive event [58] such as a lockdown by public health institutions, there are many factors that can bring about these changes, or on the contrary, not encourage them. Returning to what was stated in Section 2, reflection does not necessarily have to lead to a change in habits. In addition, as Camargo explained, a multitude of situations both at a supra-individual and individual level can affect the way of dealing with these disruptive events and therefore potentially abandon habits and/or create new ones [19]. In this case, possible causes related to personal and household situations have been evaluated, to understand, at a general level, what has happened in the lives of the confined people [38,59].

In turn, both the imposition or social norm given by the State of Alarm, as well as the adaptive behavior to a greater or lesser extent of people inside the home, has given rise to a series of new activities that could lay the foundations or consolidate new habits. These can have direct economic, social, community implications, such as ways of enjoying the city and common spaces in buildings, teleworking, e-commerce, or the way of relating, where social networks and internet connection have also certainly had a relevant place [60]. The situation of uncertainty in the face of what is to come, the risks associated with the illness itself, isolation, or fear of change for oneself or for our loved ones in any of the areas of life have also generated unstable psychological states, anxiety, stress or depression [61,62,63]. Other impacts, related to the environment, have also been given by the way we behave, such as those derived from the use of energy in the home [40,64], or the environmental ones, which have been positive during this time for the planet, cleaning our cities of greenhouse gas emissions, for example [65].

To the best of our knowledge, this is the first study to be carried out at a national level for the Spanish population on the permanence in the home and the change in habits and routines, temporary dedication to tasks and going out of the home, as the ultimate objective of the research, in relation to the composition of the household and socio-demographic data. This analysis reveals behavioral differences in terms of gender, age, and household composition, where cohabitants with a certain degree of dependency, such as children or the elderly, and their special vulnerability to the coronavirus, have largely conditioned the dedication of the adults in charge. The role of women as caregivers and home maintainers has been more pronounced, also affecting, to a large extent, their predisposition to alter health-related habits, contact with the outside world and their routines, which is in line with similar studies [66,67]. These factors can be taken into account for future contingency plans, time management strategies, or formulas that favor both equal time distribution within the household as well as exchanges and design of safe areas and other measures that provide families with support in similar situations, thereby mitigating the impact on their daily lives without detriment to the safety of cohabitants in terms of virus transmission.

## 6. Limitations

This research is not without certain limitations. In the first place, derived from the selection of the sample, by convenience, both the means of contact (social networks, websites, and other contacts), and the type of questionnaire required an internet connection and digital resources and skills to be able to answer it. Additionally, the sample showed a significant tendency of high time dedication to teleworking, which could also be due to the type of sample and the platforms selected for the dissemination of the questionnaire. It is assumed that vulnerable segments of the population were not specifically covered in this study, such as elderly people living alone, for instance, or their specific situations. Only those capable of access to the Internet and willing to answer the questionnaire were considered in this study.

Conversely, the limitation of the alteration of habits and routines could have been complemented by an assessment of them, in order to have a complete picture in relation to the socio-demographic variables of the study for the sample. In addition, the way in which some of these questions were formulated in this questionnaire were qualitative, preventing a comprehensive idea of what was happening with routines and habits of confined people. Finally, routines and habits were used in a secular sense, having not exactly the same approach that was offered in Section 2.

Nonetheless, it seemed relevant and opportune to carry out an exploratory study to assess permanence in the home, the obligation to combine work and work-life balance, and the use of spaces by cohabitants. Observing which segments of the population have seen their habits and customs altered, including going outside (mainly due to force majeure), and their occupations, could be a good way of approaching the reality experienced during lockdown in order to be able to establish preventive and contingency measures for disruptive and extreme situations such as this or similar ones in the future.

## 7. Conclusions

In conclusion, the impact of changes in habits during the general confinement of the COVID-19 pandemic seemed to fall more heavily on women, as well as on people living with children and those aged 35–54 years, especially with regard to tasks related to home care or their cohabitants. These changes in habits generally affected both the establishment of new routines, going out of the home, time dedication to tasks, and the perceived alteration of pre-pandemic habits. People over 55 years of age and those living with people over 65 were the least likely to have altered these aspects of their daily life in confinement.

## Figures and Tables

**Table 1:** Variables grouped by categories used in this study, main questions and possible answers.

Category	Variables
Socio-demographic factors	Age, gender, employment status, education level, place of birth.
Household composition	Number of cohabitants, also distinguishing presence of minors or elderly in charge.
Establishing routines	Q: Have you established a daily routine in this period of confinement? R: No routine; barely a routine; trying a routine; similar but more flexible routine; same routine (Likert scale)
Task dedication	Q: Please indicate, from 1 to 5 (1: minimum value, 5 maximum) the dedication to any of the following tasks (if you may have combined tasks in the same proportion, you can repeat scores): R: Rest, watching TV/reading, housework, caring for minors/dependents, and leisure/sports.
Going outside	Q: During the lockdown: how often are you going out? R: Never, almost never—occasionally, and frequently (which is the sum of almost every day, and every day).
Habit changes	Q: Indicate, of the following habits, which have been altered during confinement (you can select one or more response options) R: Work, caring for minors or other family members, cleaning the home, other domestic tasks (cooking, tidying...), dressing/changing clothes, eating, sleeping, leisure, smoking, drinking, practicing sports, and social relationships in the home.

**Table 2:** Sociodemographic characteristics of the study participants, by gender.

Variable	Total	Male	Female	*p* *
	*N* (% Column)	*N* (% Column)	*N* (% Column)	
General	1673 (100)	622 (37.5)	1038 (62.5)	
Age				<0.001
18–34	369 (22.4)	119 (19.4)	250 (24.1)	
35–54	909 (55.1)	317 (51.7)	592 (57.1)	
≥55	371 (22.5)	177 (28.9)	194 (18.7)	
Education level				0.142
Up to Secondary	51 (3.1)	25 (4.0)	26 (2.5)	
High School	272 (16.4)	94 (15.1)	178 (17.2)	
University	1335 (80.5)	502 (80.8)	833 (80.3)	
Employment status				<0.001
Civil servant	597 (47.9)	194 (40.8)	403 (52.3)	
Employee	387 (31.1)	148 (31.1)	239 (31.0)	
Self-employed/Entrepreneur	262 (21.0)	134 (28.2)	128 (16.6)	
Place of birth				0.008
Spanish	1543 (93.2)	55 (91.1)	57 (94.5)	
Foreign	112 (6.8)	564 (8.9)	979 (5.5)	
Cohabitants				0.946
One	347 (22.7)	127 (22.5)	220 (22.9)	
Two	425 (27.8)	160 (28.3)	265(27.5)	
Three or more	755 (49.4))	278 (49.2)	477 (49.6)	
Living with people under 18				0.272
No	978 (64.0)	372 (65.7)	606 (62.9)	
Yes	551 (36.0)	194 (34.3)	357 (37.1)	
Living with people over 65				0.702
No	1306 (85.4)	486 (85.9)	820 (85.2)	
Yes	223 (14.6)	80 (14.1)	143 (14.8)	

* *p* value for the chi-square test of the relationship of the variable with gender. A *p* < 0.05 implies a significant relationship.

**Table 3:** High dedication to tasks in confinement according to socio-demographic and coexistence characteristics.

	Telework/Telestudy		Domestic Chores		Rest		TV/Reading		Care		Leisure/Sport	
	%	*p* *	%	*p* *	%	*p* *	%	*p* *	%	*p* *	%	*p* *
Age												
18–34	61.2	**0.005**	24.9	**<0.001**	33.9	**0.001**	24.0	**<0.001**	5.2	**<0.001**	17.7	**<0.001**
35–54	61.8		38.5		23.3		14.3		39.0		8.7	
≥55	51.6		20.8		24.2		18.2		7.9		7.3	
Gender												
Male	62.3	0.083	25.0	**<0.001**	24.7	0.428	17.7	0.905	19.4	**<0.001**	9.9	0.612
Female	57.7		34.8		26.5		17.4		28.9		10.7	
Education level												
Graduated	62.7	**<0.001**	28.1	**<0.001**	24.2	**0.003**	14.8	**<0.001**	24.6	0.152	9.5	**0.027**
Undergraduate	43.8		44.4		32.5		29.1		29.0		13.9	
Cohabitants												
One	58.9	0.752	24.1	**<0.001**	27.6	0.026	19.1	**<0.001**	2.6	**<0.001**	14.6	**0.009**
Two	61.3		27.6		29.8		22.6		11.0		9.6	
Three or more	59.4		36.5		22.9		13.0		41.5		8.6	
Living with people under 18												
No	61.0	0.201	24.8	**<0.001**	29.1	**<0.001**	20.8	**<0.001**	3.7	**<0.001**	12.3	**0.001**
Yes	57.6		42.5		20.2		10.5		58.3		6.8	
Living with people over 65												
No	60.9	**0.028**	32.5	**0.009**	25.5	0.440	15.6	**<0.001**	26.6	0.095	10.3	0.098
Yes	52.7		23.6		28.0		25.3		20.6		10.3	

* *p* value for the chi-square test of the relationship with high dedication to different tasks. A *p* < 0.05 implies a significant relationship. Significant relationships are highlighted by bold *p*-values.

**Table 4:** Perceived habit changes during confinement according to the different tasks, related to socio-demographic and coexistence characteristics.

	Telework		S. Relations		Leisure		Getting Dressed		Domestic		Cleaning		Care of Minors		Sport		Sleeping		Eating		Alcohol Consumption		Tobacco Consumption	
	%+	*p* *	%+	*p* *	%+	*p* *	%+	*p* *	%+	*p* *	%+	*p* *	%+	*p* *	%+	*p* *	%+	*p* *	%+	*p* *	%+	*p* *	%+	*p* *
Age		**<0.001**		0.028		**<0.001**		**<0.001**		**<0.001**		**<0.001**						**<0.001**		**<0.001**		**<0.001**		0.560
18–34	68.0		60.3		22.4		48.6		40.5		33.8		11.9	**<0.001**	55.4	**<0.001**	44.6		28.9		14.9		6.8	
35–54	73.3		67.7		29.2		38.4		49.7		48.7		44.0		55.6		36.0		25.1		10.5		6.9	
≥55	54.4		67.8		14.2		27.6		32.7		36.2		23.3		43.4		24.1		10.2		5.9		3.5	
Gender		0.437		**0.001**		**<0.001**		**<0.001**		**<0.001**		**<0.001**		**0.018**				**<0.001**		0.011		0.598		0.083
Male	66.6		61.1		19.0		30.5		37.8		34.2		28.6		53.1	0.930	28.5		19.3		10.9		4.8	
Female	68.4		69.2		27.5		43.0		47.3		47.5		34.2		53.3		39.1		24.7		10.1		6.9	
Study level		**<0.001**		**<0.001**		0.564		**0.001**		**<0.001**		**<0.001**		**0.002**				0.320		0.502		0.831		**0.002**
Graduated	71.4		69.0		24.6		40.2		46.8		44.7		33.9		56.0	**<0.001**	34.6		23.0		10.4		5.2	
Undergraduate	52.6		53.5		23.1		29.8		31.4		32.9		24.9		41.8		37.5		21.2		10.8		9.8	
Place of birth		**0.020**		**0.013**		0.200		0.052		**0.019**		**0.001**		**0.001**				0.826		0.664		0.361		0.225
Spanish	68.5		66.7		24.6		39.0		44.6		43.6		33.1		53.8	0.132	35.2		22.8		10.6		6.3	
Foreign	57.9		55.3		19.3		29.8		33.3		28.1		17.5		46.5		34.2		21.1		7.9		3.5	
Employment status		**0.019**		0.647		0.868		**0.003**		**0.007**		**0.026**		0.440		0.069		0.061		0.632		0.155		0.408
Civil servant	78.5		69.3		24.3		42.8		48.3		48.7		35.0		59.0		36.2		22.7		8.8		5.7	
Employee	73.3		66.6		24.9		38.0		50.9		45.2		33.2		53.5		36.0		23.1		12.3		7.5	
Self-employed/Entrepreneur	70.1		67.5		23.1		30.6		38.8		38.8		30.6		51.5		28.4		20.1		11.9		5.2	
Cohabitants		0.067		0.972		**<0.001**		**0.025**		**0.005**		**<0.001**		**<0.001**		0.184		0.775		0.539		0.353		0.102
One	68.1		71.2		16.8		37.9		46.2		43.0		17.7		61.5		39.3		24.5		13.4		9.1	
Two	73.8		70.8		21.0		46.5		41.4		38.8		21.0		55.4		38.1		26.2		10.3		6.8	
Three or more	74.6		71.4		33.6		39.6		51.1		51.2		50.0		56.6		37.1		23.3		10.9		5.7	
Living with people under 18		**0.005**		**0.013**		**<0.001**		0.182		**<0.001**		**<0.001**		**<0.001**		0.595		0.607		0.739		0.126		0.456
No	70.6		69.0		20.3		42.3		41.0		39.6		17.4		57.9		38.4		24.1		12.3		7.1	
Yes	77.2		75.0		36.9		38.8		58.5		56.8		65.3		56.5		37.1		24.8		9.7		6.1	
Living with people over 65		**<0.001**		0.410		0.315		**<0.001**		**<0.001**		**<0.001**		0.547		**<0.001**		**0.001**		**0.002**		**0.030**		0.075
No	75.8		71.6		26.7		43.1		49.9		47.9		35		59.3		39.6		25.8		12.1		7.2	
Yes	56.4		68.9		23.6		29.3		32.0		33.8		32.9		45.8		28.0		16.0		7.1		4.0	

* *p* value for the chi-square test of the relationship with habit changes related to different tasks. A *p* < 0.05 implies a significant relationship. Significant relationships are highlighted by bold *p*-values.

## Data Availability

Data are not available due to ethical reasons.
